# Fecal Incontinence Drives the Psychosocial Burden in Patients with Dual Incontinence

**DOI:** 10.1007/s00192-025-06192-0

**Published:** 2025-06-20

**Authors:** Karla Rebullar, Delaney J. Orcutt, Melissa R. Kaufman, Roger R. Dmochowski, Elisabeth M. Sebesta

**Affiliations:** Vanderbilt University Medical Center, 1211 Medical Center North, Nashville, TN 37232 USA

**Keywords:** Dual incontinence, Fecal incontinence, Psychosocial burden, Urinary incontinence

## Abstract

**Introduction and Hypothesis:**

Urinary incontinence (UI) and fecal incontinence (FI) are debilitating conditions affecting patients’ quality of life (QOL). Dual incontinence (DI) of both urine and stool represents an additional burden. We compare the physical and mental health of patients with UI, FI, and DI. We hypothesize that DI might be associated with increased psychosocial burden.

**Methods:**

Patients completed online questionnaires on sociodemographics, urinary and bowel symptoms, and psychosocial comorbidity. The Patient-Reported Outcomes Measurement Information System (PROMIS) measures physical and mental health, anxiety, depression, stress, and instrumental support was used to assess psychosocial burden. Responses including PROMIS measures were compared between those with no incontinence (NI), those with UI, those with FI, and those with DI.

**Results:**

A total of 3620 respondents completed the study, the majority of whom were female (78%), and white (84%). Age and BMI were different among groups, with the DI group having the oldest mean age (54 years) and highest mean BMI (29 kg/m^2^). PROMIS scores were significantly different across groups, with DI having the lowest physical and mental health scores (i.e. poorest health), and highest stress and depression scores (i.e. greatest burden). This remains true when DI is compared only with NI or UI; however, results were similar for most measures between DI and FI, suggesting that the burden might largely be driven by FI.

**Conclusions:**

Dual incontinence results in worse patient-reported physical and mental health, and higher psychosocial burden. In our cohort, it appears that the psychosocial burden may be largely driven by the FI component of DI. Thus, addressing FI in our patients with DI may help improve QOL.

## Introduction

Urinary incontinence (UI), or involuntary leakage of urine, is a common and debilitating condition known to affect patient health and quality of life (QOL). In one study, the prevalence of UI among women in the United States was as high as 53% [1]. Fecal incontinence (FI), characterized by the uncontrolled passage of solid or liquid stool, is a similarly distressing condition associated with poor QOL [2]. In a recent report from the United States National Health and Nutrition Examination Survey (US NHANES) looking at FI prevalence from 2005 to 2010, Ditah et al. reported an overall prevalence of 8.4% [3].

Patients who suffer from any form of incontinence avoid social situations, lose confidence, and experience impairment of sexual function and mental health [4, 5]. In addition to psychosocial ramifications, incontinence represents a huge financial and economic burden, with up to 32 billion dollars a year estimated to be spent on its management in the US alone [7].

Not surprisingly, the presence of both UI and FI in the same patient, or dual incontinence (DI), represents an additional burden, and further reduces functional status and QOL. In one study, DI is estimated to be present in 20% of women presenting with UI [8].

Although it is known that UI, FI, and DI significantly impair patient QOL, less is known about the effect of each incontinence on the psychosocial burden of patients with DI. This study is aimed at comparing the physical and mental health of patients with UI, FI, and DI. We hypothesize that the presence of DI is associated with increased psychosocial burden than UI or FI alone.

## Materials and Methods

This is a secondary analysis from an Institutional Review Board-approved (#211,445) study on urinary symptoms and social determinants of health. Participants were recruited electronically via an online recruitment database, ResearchMatch, between September 2021 and January 2022 to complete questionnaires on sociodemographics, urinary and bowel symptoms, and psychosocial comorbidity [9]. Approximately 140,000 participants were contacted via a single email advertisement with an invitation to complete anonymous, electronic questionnaires using Research Electronic Data Capture (REDCap) [10]. Participants were incentivized to participate with the chance to win one of ten $100 gift cards. A total of 5233 (3.7%) people responded to the email and started the survey. Participants were excluded if they were under the age of 18, did not complete the entire questionnaire, were currently pregnant, had a prior radical/simple cystectomy, or had any form of ostomy (urine/stool). Patients who are catheterizing, or with a diagnosis of neurogenic lower urinary tract disease, multiple sclerosis, spinal cord injury, or stroke were also excluded. The final analytic sample included 3620 participants, or 78.7% of those who responded to the recruitment email.

Urinary symptoms were assessed using the International Consultation on Incontinence Questionnaire-Urinary Incontinence Short Form (ICIQ-UI SF). UI was defined as an answer of more often than “Never” to the question “How often do you leak urine?” FI was assessed via the question “How often in the past month have you experienced bowel accidents or leakage with mucus, liquid stool, and/or solid stool?”, with a positive response as more often than “Never.” DI was defined as the presence of both UI and FI in the same individual according to the above definitions. The Patient-Reported Outcomes Measurement Information System (PROMIS) four-item measures for stress, anxiety, and instrumental support, and the two-item measures for physical and mental health (derived from the global health measure). Depression was assessed using a single question to avoid redundancy. The PROMIS instruments are scored using item-level calibrations through the PROMIS Assessment Center Scoring Service. A T-score is standardized with a mean of 50, and a 10-point standard deviation calibrated to the general US population. Because standardized scoring of the stress measure is not yet available in adults, the raw score was used. For anxiety, stress, and depression, higher scores represent more severe symptoms. For physical and mental health and instrumental support, higher scores indicate better health or support.

Participants also self-reported age, race/ethnic identifier, gender identity, height, weight, highest level of education, employment status, household income and occupants living in the home, type of insurance use, and unmet social needs using a social needs screener derived from the Health Leads’ screening toolkit [11]. Responses obtained were dichotomous. For analysis, participants were categorized into groups reporting none, 1, 2, or 3 or more unmet social needs, as in previous studies [12]. To determine the poverty rate, the total household income was adjusted to the number of persons reported to be living in the household as recommended by the 2021 US Poverty Guidelines [13].

### Statistical Analysis

Descriptive statistics were performed summarizing data as counts and percentages or means and standard deviations (or median and interquartile range). Baseline sociodemographics and psychosocial outcomes were compared between those with no incontinence, those with UI only, those with FI only, and those with DI using Chi-squared testing or Kruskal–Wallis rank test. To assess for differences between the DI group and each of the other groups, pairwise comparisons using Wilcoxon-rank sum test with Bonferroni correction were performed. Multivariable linear regression was used to assess for associations between DI and psychosocial outcomes. Each model was adjusted for multiple covariates including age, body mass index (BMI), race/ethnicity, gender, education level, employment status, insurance status, and number of unmet social needs. All analysis was performed in Stata 17.0, with a *p* value of less than 0.05 considered significant.

## Results

A total of 3620 were included in the final analytic sample, including 39.2% with no incontinence of either type (*n* = 1418), 37.9% with UI only (*n* = 1373), 6.9% with FI only (*n* = 251), and 16.0% with DI (*n* = 578). All baseline sociodemographic characteristics and comparisons between the groups can be seen in Table 1. The majority identified as female (2828 out of 3620, 78%), and white, non-Hispanic (3025 out of 3620, 84%). Age and BMI were statistically different between groups, with the DI group having the oldest mean age (54 years) and highest mean BMI (29 kg/m^2^). Those with any type of incontinence had lower levels of educational attrition, were more often retired or on disability benefits, and less often reported using private insurance than those with no incontinence. The DI group had the highest percentage of patients reporting the most (3+) unmet needs (86 out of 578, 15%), and the lowest percentage of patients reporting no unmet needs (326 out of 578, 56%).

**Table 1:** Baseline sociodemographic information for all study participants, compared between those with no incontinence, urinary incontinence only, fecal incontinence only, or dual incontinence

	No incontinence	Urinary incontinence only	Fecal incontinence only	Dual incontinence	*p* value
Number	1,418 (39.2)	1,373 (37.9)	251 (6.9)	578 (16.0)	
Mean age, years (SD)	42.4 (16.9)	51.8 (16.1)	42.1 (17.5)	54.4 (16.2)	0.0001
Mean BMI, kg/m^2^ (SD)	26.1 (5.9)	28.5 (7.0)	26.7 (6.2)	29.2 (7.3)	0.0001
Gender (%)					< 0.0001
Male	350 (24.7)	178 (13.0)	75 (29.9)	94 (16.3)	
Female	1,024 (72.2)	1,166 (84.9)	167 (66.5)	471 (81.5)	
Transgender/nonbinary	42 (3.0)	27 (2.0)	8 (3.2)	10 (1.7)	
Unspecified	2 (0.1)	2 (0.2)	1 (0.4)	3 (0.5)	
Race/ethnicity (%)					0.001
White, non-Hispanic	1,149 (81.0)	1,175 (85.6)	205 (81.7)	496 (85.8)	
Black, non-Hispanic	69 (4.9)	81 (5.9)	12 (4.8)	26 (4.5)	
All Hispanic	69 (4.9)	36 (2.6)	10 (4.0)	19 (3.3)	
Asian	74 (5.2)	34 (2.5)	9 (3.6)	11 (1.9)	
Multiracial	35 (2.5)	25 (1.8)	9 (3.6)	14 (2.4)	
Other	14 (1.0)	17 (1.2)	3 (1.2)	10 (1.7)	
Unspecified	8 (0.6)	5 (0.4)	3 (1.2)	2 (0.4)	
Education level (%)					0.039
High school diploma or less	204 (14.4)	243 (17.7)	42 (16.7)	106 (18.3)	
Associate's or Bachelor's degree	646 (45.6)	571 (41.6)	122 (48.6)	259 (44.8)	
Graduate or professional degree	568 (40.1)	559 (40.7)	87 (34.7)	213 (36.9)	
< U.S. 2021 poverty guideline (%)	86 (6.1)	57 (4.2)	14 (5.6)	27 (4.7)	0.131
Employment status (%)					< 0.0001
Working full time	782 (55.2)	625 (45.5)	141 (56.2)	231 (40.0)	
Working part time	207 (14.6)	153 (11.1)	33 (13.2)	72 (12.5)	
Unemployed	86 (6.1)	76 (5.5)	17 (6.8)	31 (5.4)	
Retired	214 (15.1)	358 (26.1)	33 (13.2)	164 (28.4)	
Disability	32 (2.3)	81 (5.9)	13 (5.2)	56 (9.7)	
Other	97 (6.8)	80 (5.8)	14 (5.6)	24 (4.2)	
Health insurance (%)					< 0.0001
Uninsured	118 (8.3)	70 (5.1)	27 (10.8)	29 (5.0)	
Medicaid	83 (5.9)	92 (6.7)	18 (7.2)	45 (7.8)	
Medicare	111 (7.8)	186 (13.6)	23 (9.2)	92 (15.9)	
Private	943 (66.5)	734 (53.5)	152 (60.6)	264 (45.7)	
Medicaid/Medicare + private	80 (5.6)	153 (11.1)	18 (7.2)	76 (13.2)	
Military	19 (1.3)	29 (2.1)	2 (0.8)	6 (1.0)	
Other	67 (4.5)	109 (7.9)	11 (4.4)	66 (11.4)	
Number of unmet social needs					< 0.0001
None	994 (70.1)	946 (68.9)	145 (57.8)	326 (56.4)	
1	242 (17.1)	230 (16.8)	60 (23.9)	122 (21.1)	
2	95 (6.7)	86 (6.3)	16 (6.4)	44 (7.6)	
3+	87 (6.1)	111 (8.1)	30 (12.0)	86 (14.9)	

All psychosocial outcomes can be seen in Table 2. The DI group had a very high psychosocial burden across all measures assessed, with the lowest physical and mental health scores (i.e., poorest health), and highest stress and depression scores (i.e., greatest burden) across all groups. Compared with those with no incontinence, those with DI had significantly worse physical and mental health, and worse scores on all psychosocial measures. When comparing those with DI and those with UI or FI only, the results remained different between the UI and DI groups but were similar in the FI and DI groups for all measures except for the physical health score. On multivariable linear regression, after controlling for covariates, having DI was associated with worse overall physical and mental health (beta coefficient −2.12, 95% CI −2.76 to −1.48, *p* < 0.0001 and −1.71, 95% CI −2.43 to −0.99, *p* < 0.0001). Additionally, DI was associated with a greater burden of anxiety (beta coefficient 2.13, 95% CI 1.44 to 2.83, *p* < 0.0001), stress (beta coefficient 0.91, 95% CI 0.60 to 1.21, *p* < 0.0001), and depression (beta coefficient 0.15, 95% CI 0.05 to 0.24, *p* = 0.003). Associations between instrumental support were no longer significant.

**Table 2:** Mean PROMIS measure scores based on the type of incontinence

PROMIS Questionnaire	No Incontinence	Urinary incontinence	Fecal incontinence	Dual incontinence	*p* value^‡^
Physical Health 2a T-score (SD)	51.5 (7.5)	48.6 (8.5)	49.7 (8.1)	45.4 (8.7)*^†	< 0.001
Mental Health 2a T-score (SD)	48.2 (9.1)	47.9 (9.2)	46.4 (8.9)	45.6 (8.8)*^	< 0.001
Anxiety 4a T-score (SD)	53.6 (9.3)	53.2 (9.4)	55.8 (9.0)	55.7 (9.5)*^	< 0.001
Stress 4a raw score (SD)	9.4 (4.2)	9.1 (4.2)	10.3 (4.3)	10.3 (4.4)*^	< 0.001
Depression raw score (SD)	2.1 (1.1)	2.2 (1.2)	2.3 (1.2)	2.4 (1.2)*^	< 0.001
Instrumental support 4a T-score (SD)	51.8 (10.6)	51.9 (10.2)	49.1 (10.7)	50.4 (10.3)*^	< 0.001

*SD* standard deviation

^‡^ Kruskal–Wallis rank test

Significant *p* value < 0.05 on pairwise comparison using a Wilcoxon rank-sum test with a Bonferroni correction:

^*^Compared with the no-incontinence group

^Compared with the urinary incontinence-only group

^†^Compared with the fecal incontinence-only group

## Discussion

In a large community-based sample, we observed that those with DI consistently had a significantly greater psychosocial burden, even after adjusting for other covariates, including a poorer perception of physical and mental health, and higher burden of stress, anxiety, and depression. Patients with DI reported significantly worse physical health, mental health, anxiety, stress, and depression than patients with UI only. The DI and FI groups significantly differed in physical health scores only.

In our study population, DI patients were older and had a higher BMI than patients with UI or FI only. Similarly, in a cross-sectional study identifying risk factors associated with incontinence, Wu et al. found that the prevalence of DI increased with age in both sexes [14]. Similar conclusions have been drawn from studies evaluating women alone [15]. As patients age, the integrity of connective tissues and neuromuscular function decline whereas the number of comorbid illnesses such as stroke, diabetes, cognitive impairment, and immobility increases. Control of urine and stool depends on proper function and coordination of central and peripheral mechanisms; therefore, it is unsurprising that advanced age is associated with loss of both bladder and bowel control.

The DI group also had the highest proportion of patients reporting higher unmet social needs. Social needs include domains such as housing quality and instability, food insecurity, utilities, health care costs, child care, interpersonal relationships, and transportation challenges [12]. Recent studies from our group investigating the relationship between unmet social needs and severity of multiple noncancerous genitourinary conditions found that those with three or more unmet needs were at a higher risk of urinary conditions such as UI and overactive bladder [12, 16]. To our knowledge, there is no dedicated study in the literature assessing the relationship between FI or DI and patients’ unmet social needs. Future research in this area can be of great value in the overall management of DI, as addressing these unmet social needs can help to improve care.

On multivariable linear regression controlling for covariates, our study found DI to be associated with a greater burden of stress, anxiety, and depression. Although depression has been shown to be more strongly associated with DI than with FI or UI in other studies [14], to our knowledge this is the first study to report a correlation between DI, anxiety, and stress. Another large study evaluating patients with UI versus DI found no difference in the risk of depression between the two groups [17]. In contrast, Wu et al. found that moderate-to-severe depression was significantly associated with DI, with an odds ratio of 4.7 [18]. There are case reports in the literature describing DI with no identified organic cause in patients presenting with depression [19, 20]. They describe the hypothesis that mood disorders and incontinence may be a manifestation of changes in the frontal lobe of the brain. Further research is necessary to explore the prevalence and relationship of DI in mood disorders. The relationship between mood disorders and DI is likely bidirectional; therefore, providers should strongly consider screening for concomitant anxiety and depression in patients presenting with DI and connecting patients to mental health services when appropriate to help manage their overall QOL.

Our study found that those with DI had significantly worse physical health, mental health, and psychosocial scores across all groups. This statistically significant difference persisted on pairwise comparisons when comparing patients with DI and patients with UI, but not when comparing patients with DI and patients with FI. In a similar study, Fialkow et al. reported worse functional status and QOL in women with DI [21]. Although their study suggested the additional effect of FI to the patients’ perception of increased severity of their UI, there was no comparison of responses between FI and DI groups. Therefore, unlike in our study, no inferences were made regarding the effect of the presence of FI on the DI population. Our study suggests that psychosocial outcomes were similar in the FI and DI groups for all measures except for the physical health score. Kravitz et al. evaluated the association between DI and fall risk and found that there was a greater risk of falls in women with DI than in women with UI alone, further highlighting the negative effect of FI in addition to UI, and the greater vulnerability of this population [17]. In their study evaluating UI and DI in older women after hip fracture, Hellman-Bronstein et al. observed that factors associated with DI but not UI included institutional living, non-independent mobility, and poor nutrition [22]. The retention of statistical significance when comparing responses of the DI group with those of the UI group (i.e., patients with DI continued to have worse health and a greater burden than those with UI) but not when comparing with those of the FI group (i.e., no difference) suggests that the FI component could be driving the psychosocial burden in patients with DI.

Limitations to this study includes its cross-sectional design; therefore, causal relationships cannot be established. Recruitment of participants online from ResearchMatch also introduces selection bias, as reflected in the homogenous study population consisting of mostly white, non-Hispanic females. Although the eventual response rate was high at 78.7% (3620 out of 5233), the initial response rate was low (5233 out of 140000, 3.7%). Collectively, these limit our study’s generalizability to a more representative population. Additionally, the study is prone to recall bias as we relied on patient responses to identify symptoms, and, owing to the nature of our anonymous questionnaire, we do not have the ability to confirm patient-reported responses. We did not examine the severity of urinary or bowel symptoms for the purpose of this study; however, other differences may have been observed depending on the severity of UI, FI, or both. Moreover, we neither reported on nor performed any analysis involving the parity (and mode of delivery) of the women in our study. Previous studies have shown significant associations between vaginal deliveries and pelvic floor disorders including stress incontinence and FI [23, 24]. It should therefore be noted that the differences in the proportion of multiparous women with vaginal deliveries in each group (NI, UI, FI, DI) can affect the study findings.

Despite these limitations, this is one of the largest study populations used to examine the impact of DI on psychosocial burden, and to compare with UI and FI alone in a community-based cohort. Our study supports the hypothesis that patients with DI have worse physical and mental health than those with either UI or FI in isolation. In addition, to our knowledge, this is the first study to compare FI and DI and show a lack of significant difference in patient-reported outcomes, potentially indicating that FI may be the driving force of worse psychosocial burden and health scores in patients with DI. In our cohort and in previous studies, the proportion of patients with DI is approximately 18–20%, indicating that UI and FI often coexist [8, 21]. Our study suggests that prioritizing management of the FI in these patients may improve their QOL more significantly than if the UI component were addressed first.

## Conclusions

Unsurprisingly, DI results in worse patient-reported physical and mental health, and a greater psychosocial burden. In our cohort, it appears that the psychosocial burden in patients with DI may be largely driven by the FI component. This finding may help to tailor treatment toward undertaking the FI issues in patients with DI primarily to improve QOL in these patients. Future directions involve reassessment of QOL via similar PROMIS questionnaires after management of FI in patients with DI, and further exploring the relationship between DI, FI, and unmet social needs.
